# Current concepts in the techniques, indications and outcomes of meniscal repairs

**DOI:** 10.1007/s00590-018-2317-5

**Published:** 2018-10-29

**Authors:** Monil Karia, Youssef Ghaly, Nawfal Al-Hadithy, Simon Mordecai, Chinmay Gupte

**Affiliations:** 1 Musculoskeletal Lab, Imperial College London, London, United Kingdom; 2 Orthopaedic Department, Hillingdon Hospital, London, United Kingdom

**Keywords:** Meniscal repair, Trauma, Management, Arthroscopy, Knee

## Abstract

Knee arthroscopy for meniscal tears is one of the most commonly performed orthopaedic procedures. In recent years, there has been an increasing incidence of meniscal repairs, as there are concerns that meniscectomy predisposes patients to early osteoarthritis. Indications for meniscal repair are increasing and can now be performed in older patients who are active, even if the tear is in the avascular zone. Options for meniscal tear management broadly fall into three categories: non-operative management, meniscal repair or meniscectomy. With limited evidence directly comparing each of these options optimal management strategies can be difficult. Decision making requires thorough assessment of patient factors (e.g. age and comorbidities) and tear characteristics (e.g. location and reducibility). The purpose of this paper is, therefore, to review the management options of meniscal tears and summarize the evidence for meniscal tear repair.

## Introduction

Meniscal tears are one of the most common knee injuries and often necessitate surgery due to pain or mechanical symptoms. Historically, menisci were considered vestigial remnants and were commonly resected. In 1948 Fairbank [1] suggested that meniscectomy predisposed the knee to early degenerative changes, and there have since been several studies confirming poor long-term function and early degenerative changes in patients post meniscectomy. In more recent years various important roles of menisci including load sharing, shock absorbers, secondary stabilizers, proprioception and lubrication have all been confirmed [2].

The first meniscal repair was performed by Annandale in 1885 with an open procedure and despite development of various arthroscopic techniques in the subsequent years; it is only recently that meniscal preservation has received a high level of awareness. In 2013, a review of arthroscopic procedures found a doubling in the number of meniscal repairs performed in the past 5 years, without a concomitant increase in meniscectomies [3]. It is now accepted that knees where there has been a meniscal repair have lower rates of radiographic degenerative changes compared with meniscectomy. Last year, a systematic review of various repair techniques and implants was performed and found no difference in outcomes, however focused on outcomes greater than 5 years, which therefore excluded many of the newer all-inside repair techniques [4].

There is still controversy and uncertainty as to the ideal management of meniscal tears, including which meniscal tears should be repaired, methods of assessment post repair, and the effect of damage to the anterior cruciate ligament (ACL). Lastly a summary of the clinical outcomes for various techniques and devices is presented.

## Blood supply

**Fig. 1:**
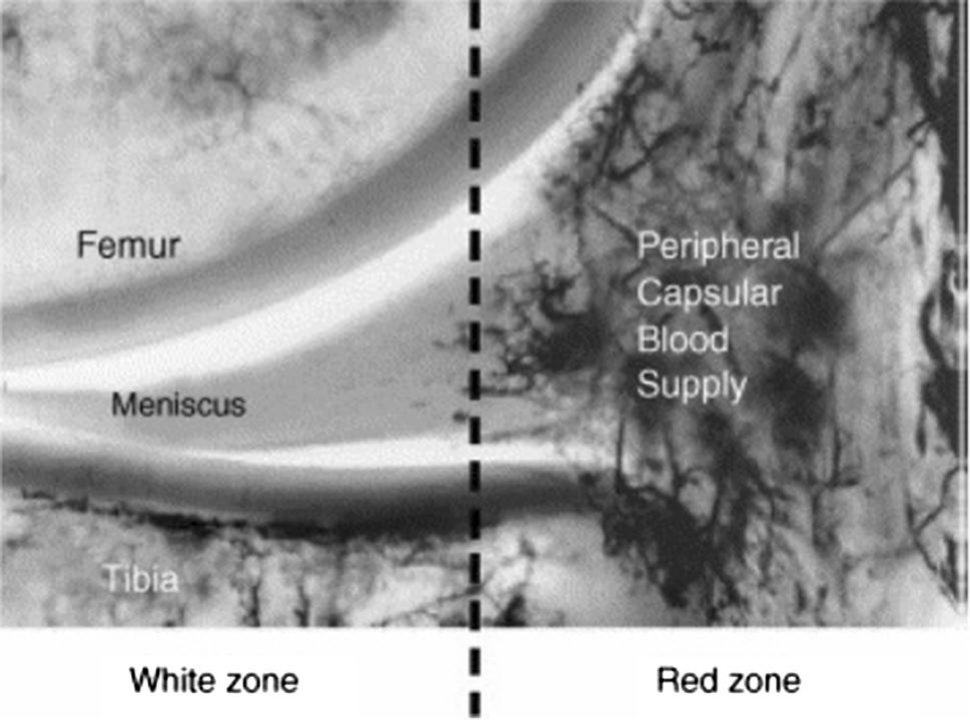
Radial branches penetrating the peripheral ¼ of the lateral meniscus

**Fig. 2:**
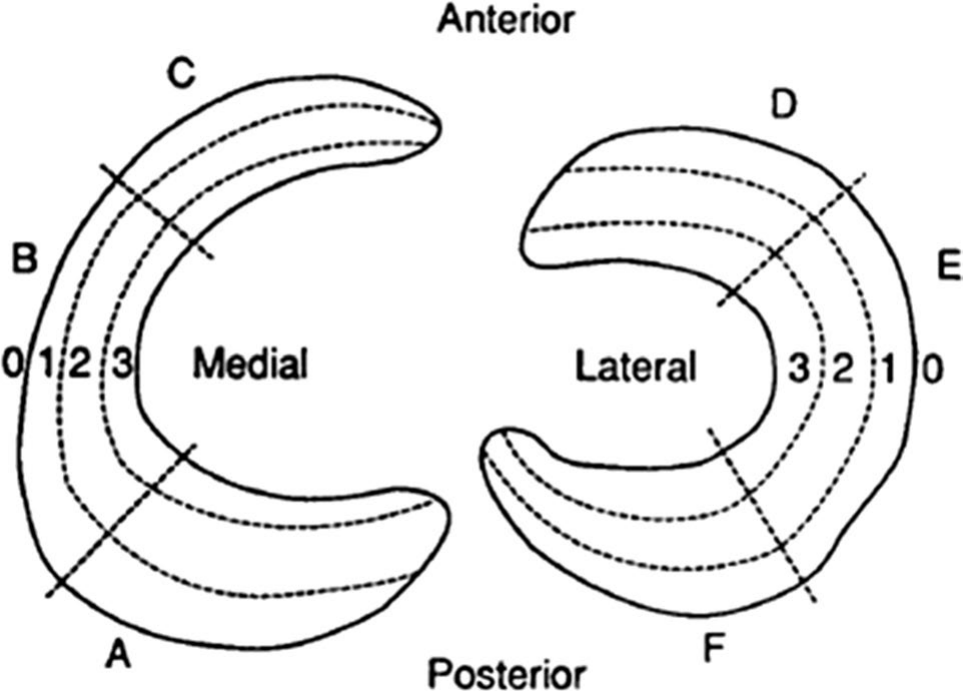
Cooper zones of the meniscus

Blood vessels arise from the lateral, middle and medial geniculate arteries and penetrate through the joint capsule to form a perimeniscal capillary plexus, where radial branches enter the menisci and supply the peripheral quarter of the menisci (red zone). In cadaveric studies, Arnoczky et al. [5] and Day et al. [6] found that radial branches penetrate the menisci to a depth of 2–3 mm (Fig. 1), with the most consistent blood supply occurring at the anterior and posterior horns. Both studies found that the posterolateral aspect of the lateral meniscus, adjacent to the popliteus tendon was avascular as was the inner 70–75% of the menisci (white zone). Cooper described these zones by dividing the meniscus into 3 radial sections (Zone A, B and C) from posterior to anterior and the width into 3 from peripheral to central (Fig. 2).

In addition, the blood supply to the menisci varies with age. Petersen and Tillmann [7] reviewed cadaveric specimens of 20 human menisci ranging in age from birth to 80 years old and found that at birth the whole meniscus was vascularized. By the second year, they had an avascular area on the inner circumference. By the age of 20, blood vessels were only present in the peripheral third, which further regressed to a quarter at the age of 50.

Some authors have investigated the effects of osteoarthritis on the vascularity of menisci. Previously it was found that increased angiogenesis was present in OA synovium. More recently, Ashraf et al. [8] used antibodies to localize blood vessels by histochemistry and found an increased density of blood vessels near the fibrocartilage junction in patients with high tibiofemoral chondropathy. They suggested this, in addition to an increase in the number of perivascular sensory fibres, may be possible mechanisms contributing to knee pain in OA. This suggests why meniscal surgery may alleviate pain.

### Meniscal tear patterns

**Fig. 3:**
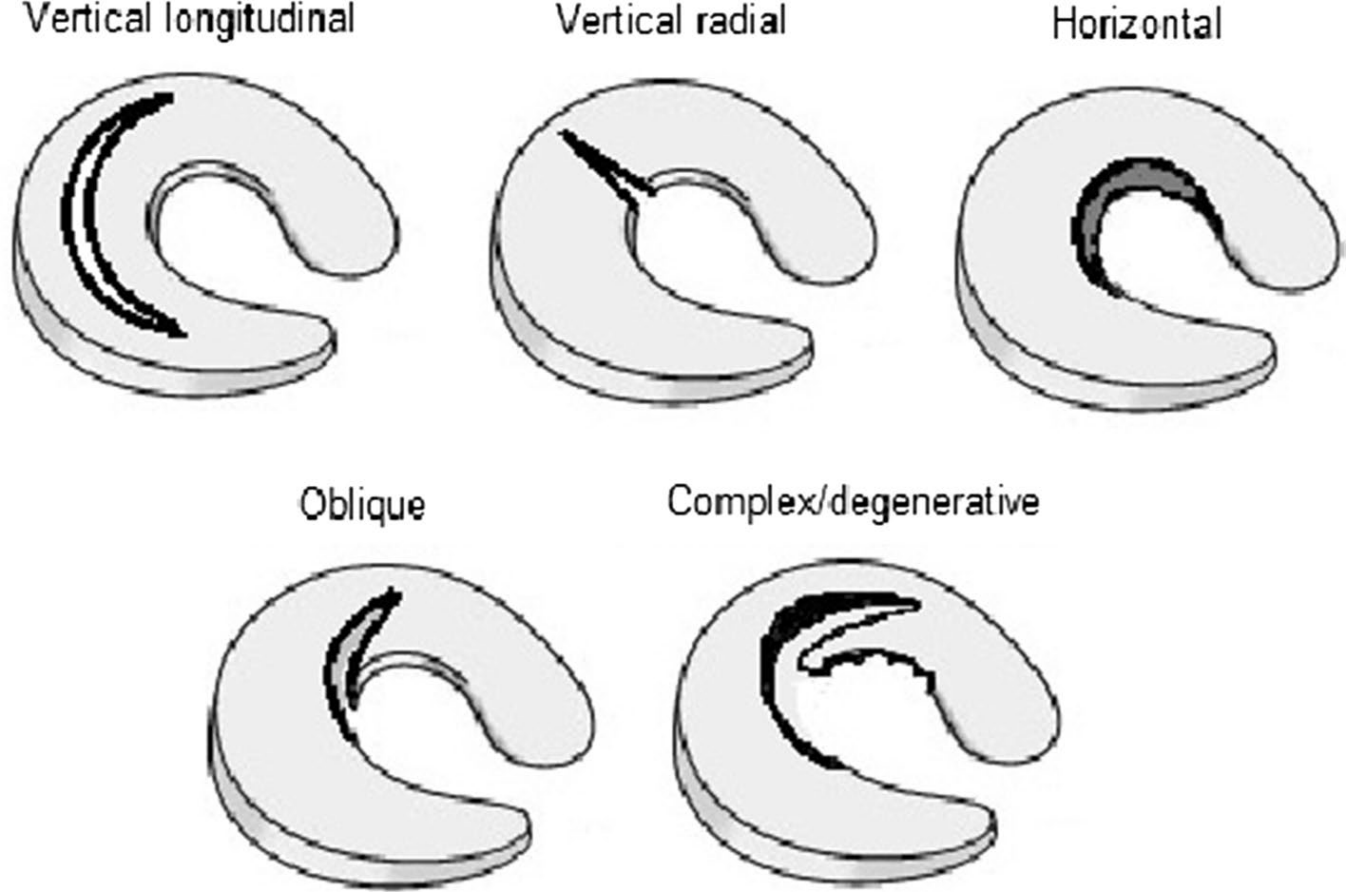
Diagram to illustrate the types of meniscal tears

Meniscal tears may be classified according to anatomic location and therefore proximity to blood supply and also tear morphology (Fig. 3). The tear characteristics vary depending on many factors including stability and sporting activity.

The pattern of meniscal injuries in the ACL-deficient knee is similar in both children and adults, with a higher but non-progressive incidence of lateral meniscal tears occurring acutely, and a lower but increasing rate of medial tears correlating with chronicity. Baker et al. [9] reviewed meniscal injuries according to specific sports and found medial tears were more common than lateral (81% vs. 19%) and were consistently more common in football, basketball, skiing and baseball, whereas lateral meniscal tears were equal to medial tears in wrestling. Terzidis et al. [10] evaluated 378 athletes with stable knees, and found 77.5% were vertical (cf 22.5% horizontal).

Vertical tears are the most common to be repaired. They are often full thickness and may be unstable (bucket handle). They usually occur in traumatic injuries and are associated with ACL ruptures. They are often extensive, and resection would equate to a subtotal excision. If the displaced fragment is not degenerative, which may be the case in chronic injuries, it should be assessed for reducibility and considered if repairable. Most of the reported clinical outcomes related to vertical tears.

The effects of radial tears depend on whether they are incomplete or complete. Incomplete tears start centrally and extend to but do not reach the intact peripheral rim; the circumferential collagen fibres of the meniscus remain intact and stability remains. Small incomplete radial tears, which are in the white–white zone, are often treated with partial meniscectomy with successful outcomes. This contrasts with complete radial tears which traverse the circumferential collagen fibres resulting in extrusion of the meniscus and abnormal load transmission which is equivalent to total meniscectomy. Ode et al. [11] performed serial sectioning and repairs of lateral meniscal radial tears in 5 cadaveric human knees and found significantly reduced contact area and increase in contact pressures in complete radial tears, which approached similar values seen in total meniscectomy. No difference was seen in lesser degrees of tears (< 75%) as compared with an intact meniscus. Although repairs of complete tears significantly improved biomechanics, it was still reduced compared with the intact meniscus. Bedi et al. [12] also found large medial meniscal radial tears (< 90%) had reduced tibiofemoral contact pressures if repaired rather than resected in their cadaveric study.

Horizontal cleavage (intrasubstance) tears extend parallel to the tibial plateau, dividing the meniscus longitudinally, and are stable. They tend to occur as part of a degenerative process and may be accompanied by a meniscal cyst and may exist without clinical symptoms [13]; due to this they tend to be resected. Biedert [14] evaluated 4 treatment options for 41 horizontal tears; conservative, repair, trephination and repair with a fibrin clot and partial meniscectomy. The best short-term outcomes (26.5 m) were with partial meniscectomy. Pujol et al. [15] suggested there is a subgroup of younger active patients who have horizontal tears due to overuse and trauma rather than as part of a degenerative process who would benefit from repair. They performed open repairs in 21 patients with a mean age of 25 and found that 20 patients were able to return to sports at 40 months follow-up. Although 4 patients (19%) required secondary meniscectomy, they suggested this outweighed the progression of degenerative changes associated with meniscectomy.

## Extent of tear

It is generally accepted that the blood supply to the meniscus is, among other factors, a key factor that determines the outcome of meniscal repairs. It had previously been thought that meniscal repairs were only successful in vascularized zones (Red–Red or Red–White). However, Rubman et al. [16] published their series of 198 repairs that extended into the avascular zone, and 80% were clinical successful with 20% requiring re-operation at 42 months. Gallacher et al. [17] performed all-inside meniscal repairs in the white on white zone and had a 68% success rate with significant gains in Lysholm scores; however, follow-up was limited at 12 months and success was determined on re-operations rate only. Twenty-eight patients required further surgery (8 re-repair, 20 meniscectomy). In a series with the longest follow-up of 16.8 years, Noyes et al. [18] also had successful outcomes in their series of 29 meniscal repairs in patients with meniscal tears extending into the central avascular region. They had 11 (38%) failures, with 6 patients requiring meniscectomies, 2 developing early onset osteoarthritis and 3 patients although asymptomatic had failed repairs on MRI evaluation. Some authors feel that unhealed menisci which remain reduced with adequate fixation contribute to load transmission and therefore protect articular cartilage. This data are summarized in Table 1.

**Table 1:** Repairs in avascular zones

Authors	No. of patients	Mean age (years)	Mean FU	ACL	Zone	MM/LM	Time from injury to repair	Technique	Evaluation method	Outcome
Rubman [16]	177	28	42 months (18 months arthroscopically)	128 ACL rupture (126 reconstruction)	R–W	106 MM 92 LM		Inside-out	161 clinical examination 91 Arthroscopy	20% required repeat arthroscopy Of 91 arthroscopy 58% further meniscal surgery
Noyes [18]	29	16.8 (10.1–21.9)	16.8 year	24 ACL reconstruction	R–W	11 MM 18 LM	31 weeks	Inside-out vertical divergent sutures		6 meniscectomies 2 early onset OA 3 failed on MRI
Gallacher [17]	87 73 M 14 F	26 (13–54)	12 months (13–115)	Intact	W–W	50 MM 37 LM 3 PT 84 FTT 69 BHT 14 PHT	8 months (1 week–10 years)	All-inside Clear fix (72) FasT-fix sutures (13) Both (2)		Lysholm 61 → 75 Tegner 6 → 6 8 re-repairs 20 meniscectomies LM = MM outcomes
Noyes [21]	29 23 M 6 F	45	33 months	21 ACL reconstruction	R–W 4 mm RW	19 MM 11 LM	10 < 10 weeks from injury 19 > 10 weeks from injury	Inside-out suture repair		3 partial meniscectomy

*W*–*W* White white, *MM* medial meniscus, *Lm* lateral meniscus, *PT* partial tear, *FTT* full-thickness tear, *BH* bucket-handle tears, *PHT* posterior horn tears, *RW* rim width

### Patients age

Several studies have found that menisci with few or no intrinsic cells are more prone to acute or degenerative tears and that the presence of viable “normal” meniscal cells is an important factor for determining meniscal survival. Mesiha et al. [19] reviewed histological characteristics of 44 meniscal tears and found decreased intrinsic and perimeniscal cellularity in patients greater than 40 years old compared with the control group. Despite this, some authors have reported successful outcomes of repairs in older patients; Barrett et al. [20] had a high early clinical success rate (86.5%) at 26.5 months in patients aged 44 years (*n *= 37). Five patients had recurrence of clinical symptoms, and further arthroscopies were offered. Noyes et al. [21] evaluated repair outcomes in patients with a mean age of 45 who underwent meniscal repair with or without a concomitant ACL reconstruction (72%) and had very good/good outcomes in 88% of patients, with 3 requiring a meniscectomy at 33-month follow-up and suggested meniscal repair should be considered in active patients regardless of age.

### Chronicity

It has been thought that early meniscal repair provides better outcomes. Nishida et al. [22] evaluated the cell count and morphology of iatrogenic bucket-handle tears in dogs at 2, 4, 12, 24 and 48 weeks. They found that the cell count and morphology remained consistent until 12 weeks, however progressively deteriorated afterwards and suggested that repair may be more successful if performed before 12 weeks. Other authors have found a direct correlation between time since injury and meniscal DNA fragmentation and adjacent cartilage degeneration. In agreement, Pujol et al. [23] found a strong relationship between time from injury and extent of tear and subsequent meniscectomy volume and suggested symptomatic meniscal tears should be operated on as early as possible. It has been well established that there is a significantly lower chance of meniscal repair as time from injury progresses.

### Associated ACL injury

It has traditionally been thought that meniscal repair was more likely to be successful if a simultaneous ACL reconstruction was performed compared with ACL-deficient and ACL-intact knees [24], due to the iatrogenic haemarthrosis caused by the drilling of the tunnels. However, this conclusion was based largely on small and short-term studies. Last year, Nepple et al. [4] performed a systematic review of mid- and long-term studies (*n *= 8) of meniscal repairs in ACL-reconstructed knees and did not find an association with more successful outcomes. However, it is important to note that of the 8 studies, only 3 directly compared outcomes, and the studies may have been underpowered to detect a difference. Recently, Wasserstein et al. [25] compared 1332 patients who underwent meniscal repair with and without ACLR at a mean age of 25.5 years using a variety of repair techniques. They found meniscal repairs performed in conjunction with ACLR had a 7% absolute and 42% relative risk reduction of re-operation at 2 years. Whilst their data are the largest published series and may be a representative of the true population, it did not account for tear location, characteristics, surgical technique and rehabilitation protocols.

### Indications and contraindications

The surgeon must take into various patient factors and tear characteristics when deciding whether to repair or resect a meniscal tear (Table 2).

**Table 2:** Indications for repair

Patient factors	Tear characteristics
Younger (< 40), active patient	Red–red/red–white—ideal but not mandatory
No significant comorbidities	Simple tear pattern
BMI < 30	< 3 months old
Willingness to comply with post-operative rehabilitation regime	Associated ACL reconstruction
	Reducible without excess tension
	Lower threshold for complete radial tears

Contraindications to repair include: the presence of grade 3–4 osteoarthritis in ipsilateral compartment, irreducibility of the tear as the meniscus would be under too high tension and a central radial tear < 25%

### Surgical repair

Meniscal repair was initially performed open, until in 1969 when Ikeuchi [26] performed the first arthroscopic repair, and since then arthroscopic techniques have evolved which include inside-out, outside-in or all-inside repairs. As the awareness for meniscal preservation has risen, so too have the number of devices that allow for an all-inside procedure to be performed which avoids the need for further incisions, reduces the risk of neurovascular injury and faster operating times.

### Open

Open meniscal repair is now less commonly used, but may still be indicated in extremely tight medial compartments to facilitate access to a complex posterior horn tear. It is performed through an incision posterior to the collateral ligaments, through the capsule and synovium to allow direct exposure to the torn meniscus. There have been various long-term studies which have had satisfactory results, albeit with small patient numbers, and show re-tear rates between 11–29% [27]. In a study with the longest follow-up, Rockborn and Gillquist [27] had a clinical failure rate of 29% at 13 years in their series of 31 patients. There was a significantly reduced incidence of degenerative changes compared with their series of meniscectomies; in addition 80% of their patients had normal function during daily activities, with a Lysholm score of > 84. They also noted an equal decline in sporting activities in their meniscal repair group as compared with their control group with unaffected knees. These data are summarized in Table 3.

**Table 3:** Summary of open meniscal repairs with long-term follow-up

Authors	No. of patients	Mean age (years)	Mean FU (years)	ACL	Zone	MM/LM	Time from injury to repair	Evaluation method	Outcome
DeHaven [52]	30	18.9	10.9	15 ACLR	R–R	23 MM 10 LM		Clinical	21% re-tear
Muellner [38]	22	32.2	12.9	7 ACLR		18 MM 5 LM	8.7 days	Radiographs MRI	9% re-tear 27% degenerative changes
Rockborn [27]	31	25	13	All intact		17 MM 14 LM	13.5 weeks	Clinical Radiographs	29% re-tear

### Arthroscopic-assisted inside-out and outside-in techniques

Many surgeons still consider the inside-out technique to be the gold standard of meniscal repair as it allows more a more consistent suture placement, perpendicular to the tear. After introduction of the arthroscopic for intra-articular evaluation, accessory posteromedial or posterolateral incisions are required for suture retrieval. In inside-out the sutures are introduced from inside the knee, with them being knotted onto the capsule. Tears of the posterior and middle thirds of the meniscus are suitable for this technique. Outside-in techniques are more suitable for repair of the anterior and middle thirds of the meniscus. Once the tear is identified using arthroscopy, the skin is transilluminated to localize the tear. A vertical mattress suture can then be fashioned to repair the torn meniscus.

These allow for safe suture tying and aim to reduce the risk of neurovascular injury. The structures at risk depend on location of the meniscal tear; lateral meniscal repairs risk lateral genicular artery and branches of peroneal nerve. With medial meniscal repairs, the saphenous vein and nerve are at risk.

## All-inside

Various all-inside devices have been used with early generations consisting of a rigid device and newer devices being suture based. One of the first all-inside arthroscopic meniscal repair device was the Meniscus Arrow (Bionx Implants, Blue Bell, PA) which consists of a rigid degradable poly-lactic acid arrow and was initially introduced in 1993 and by 1998 had 34.4% of the US market share [28]. In an early study, Gill et al. [29] had excellent results in their series of 32 patients undergoing meniscal repair with only 3 patients (9.4%) requiring further meniscal surgery. However, in their follow-up study [30], they found their success rate had deteriorated to 71.4% at 6.6 years with a mean time of recurrence of symptoms of 43 months post-repair and attributed it to incomplete meniscal healing due to degradation of the fixation device. Arnoczky et al. [31] reviewed the biomechanical strength of rigid absorbable implants and found that devices made from polydioxanone including the Mitek meniscus refixation device and Surgical Dynamics S.D.sorb staple were found to undergo hydrolysis which significantly reduced their failure strength at 12 and 24 weeks. Other rigid devices have also been introduced with similar results; however complications including chondral injuries, synovitis, implant migration and 4 fragmentation, and soft tissue irritation have caused concerns, causing some surgeons to abandon it from their practice.

**Fig. 4:**
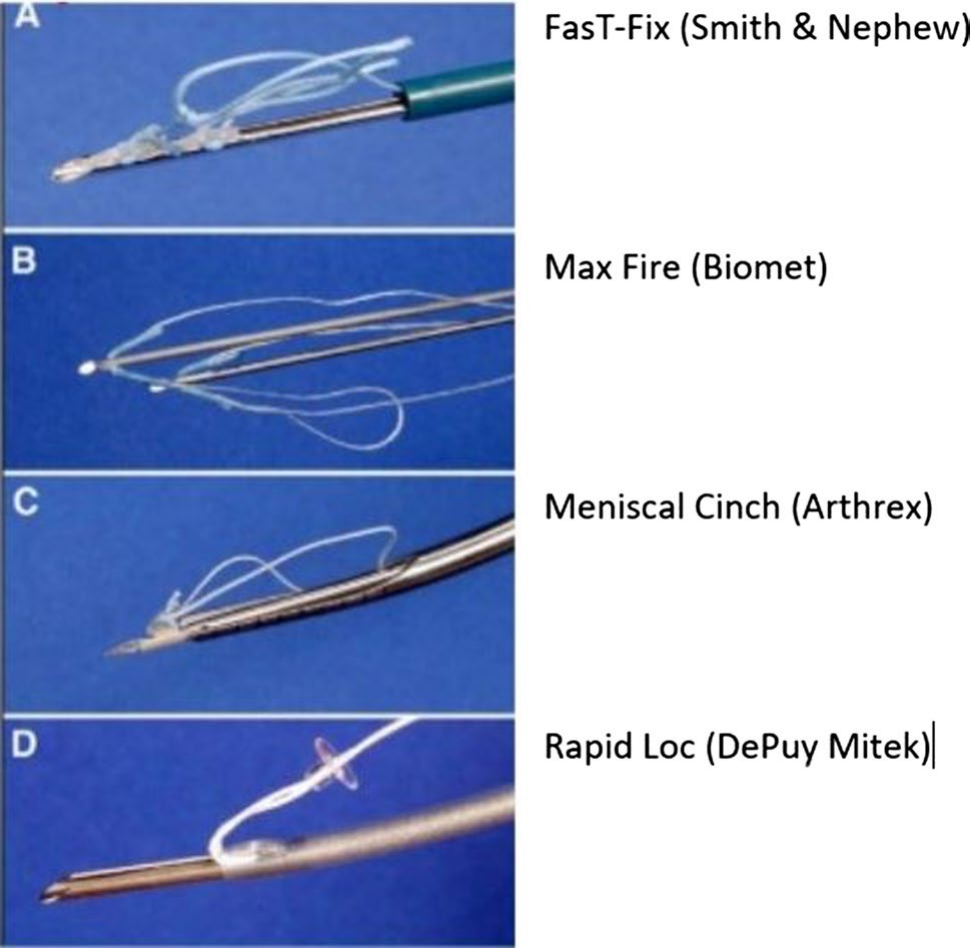
Examples of all-inside suture repair devices

In an attempt to avoid the complications associated with rigid devices and to allow more controlled tensioning, suture-based implants have been developed which consist of an anchor component and a sliding knot, which allows compression of the torn meniscal segments together examples of which are shown in Fig. 4. The FasT-Fix (Smith and Nephew Endoscopy, Andover, MA) is one such example consisting of two anchors, connected by a preloaded, pre-tied self-sliding and self-locking knot. Kotsovolos et al. [32] published their early results of 36 repairs at 18-month follow-up and had significant improvements in Lysholm scores (43.6–87.5) and had a failure rate of 12%, which was due to stiffness rather than re-tear. In a study with longer follow-up, Barber et al. [33] evaluated 41 meniscal repairs at 30.7-month follow-up and had a clinically effective meniscal repair in 83% of patients. Repeat arthroscopies were performed in 12 repairs; however, failures were only found in 7 (17%). The most common adverse event encountered was toggling and pullout of the anchors during the insertion process. In one of the few studies which evaluated the repair with a second-look arthroscopy, Tachibana et al. [34] found a clinical success rate of 83% in their series of 46 patients undergoing 65 meniscal repairs at 14 months. During arthroscopy 11 had failed and 9 had incompletely healed. There were six complications for improper deployment which needed repeat procedures.

Barber and Herbert [35] evaluated the mechanical fixation of various meniscal devices. They found that suture-based devices had a greater pullout strength than rigid devices, and others have found they approach the strength of current suture techniques. These data are summarized in Table 4.

**Table 4:** Summary of outcomes of all-inside meniscal repairs

Authors	No. of patients	Mean age (years)	Mean FU	ACL	Zone	MM/LM	Time from injury to repair	Implant	Evaluation method	Outcome
Jones [28]	38	29.9	29.7 months	22 ACLR	R–R 77% R–W 19.4% W–W 2.8%	33 MM 6 LM	5.9 months	Meniscus Arrow	Telephone questionnaire	2 partial meniscectomy 31.6% local irritative symptoms 2 removal of fragments
Gill [29]	32		2.3 years				16 acute < 6 weeks 23 chronic > 6 weeks	Meniscus Arrow	Clinical	9.4% failure rate 3 partial meniscectomy
Lee [30]	32		6.6 years	32 ACLR				Meniscus Arrow		28.6% failure rate
Siebold [53]	95	30	6 years	63 ACLR	RR R–W	77 MM 18 LM	3 months	Meniscus Arrow	Clinical	28% meniscectomy ACL had no effect Lysholm 64 → 90.5
Kurzweil [54]	57	27	54 months	42 ACLR				Meniscus Arrow	2nd look arthroscopy and MRI	28% failure rate
Kotsovolos [32]	58 37 M 21 F	32.6	18 months	36 ACLR	RR 36% R–W 64%	34 MM 27 LM	76 days	FasT-Fix	Clinical	9.8% failure Lysholm 43.6 → 87.5 4 stiff knee
Barber [55]	41 25 M 11 F	28	30.7 months	29 ACLR	R–R 37% R–W 63%	26 MM 15 LM	13 weeks	FasT-Fix	Clinical	Lysholm 47.3 → 87.4 17% meniscectomy
Tachibana [34]	46 20 M 26 W	26.5	14 months	46 ACLR	R–R 34 R–W 28	28 MM 34 LM	37 months	FasT-Fix	2nd Look arthroscopy	
Konan [51]	288	32	18 months	138 ACLR		171 LM 141 MM		54 Meniscus Arrow 258 FasT-Fix	Clinical ± MRI if clinical doubt	22.2% failure rate Meniscus Arrow 10.3% failure rate FasT-Fix 3 painful capsular sutures requiring removal

### Assessment of repair

One reason for the heterogeneity in reported outcomes is the difficulty in assessing if the repair has healed or not. Commonly used outcome measures include history and clinical examination, imaging ranging from plain radiographs (progression of tibiofemoral osteoarthritis), MR imaging or arthrogram and second-look arthroscopy. Estimations of when complete meniscal healing occurs varies between 3 and 6 months [36].

Clinical signs of an unhealed meniscal repair were described by including the presence of swelling, joint line tenderness, locking symptoms, and a positive McMurray test. However, the absence of clinical symptoms does not indicate meniscal healing, as up to 10% of patients evaluated with a second-look arthroscopy who had incompletely healed menisci were asymptomatic. The most commonly used scoring scale is the Lysholm Scale, and although during its validation process had a group of patients with meniscal pathologies, it was initially intended for multi-ligament injuries. Although Briggs et al. [37] found it to be acceptable in their evaluation into its reliability and validity, they suggested the Western Ontario Meniscal Evaluation Tool (WOMET) to be superior.

MRI is the most common radiological tool used. Although this has excellent sensitivity and specificity (~ 90%) for primary meniscal tears, some authors find its role in assessment of meniscal repairs of limited value due to the high noise–signal ratio, which can remain as high as 5.5 × the normal value even at 12 months post-operatively. Furthermore oedema and scar of the repaired site elicits a high signal and may confuse assessment; furthermore it may be present in up to 50% of cases even as long as 12.9 years post-operatively [38].

Arthroscopy remains the gold standard in evaluating meniscal repairs, as it allows the opportunity to define the length of the healed segment and probe undisplaced segments for stability of repair. Henning [39] defined incomplete healing as the persistence of a cleft at the site of the meniscal tear measuring 10–50% of the meniscal thickness. A cleft more than 50% represented an unhealed repair. Second-look arthroscopy is mostly limited to assessing and treating failed repairs. Due to its high cost and invasiveness, it is rarely used. Miao et al. [40] recently compared clinical assessment, magnetic resonance imaging and second-look arthroscopy and although clinical assessment was likely to under-estimate the healing rate (63 confirmed healed cf 77 arthroscopy), they suggested that when combined with MR, may be sufficient to exclude a re-tear.

More recently, there has been interest in MR arthrographic assessment. Magee et al. [41] found that 16 patients who underwent meniscal repair, had equivocal findings on MRI, and underwent MR-arthrography which confirmed 10/16 re-tear rate which was confirmed on second-look arthroscopy.

### Rehabilitation regimes

There are various rehabilitation regimes that range from non-weight bearing immobilization to full-weight bearing and full range of motion. The choice of rehabilitation may be influenced by meniscal tear pattern, location, repair technique and patient’s compliance. Early reports of meniscal repairs involving open procedures tended to have a greater degree of restriction with patients often kept in plaster immobilization and non-weight bearing for 4–6 weeks with a gradual increase in range of motion in the subsequent months. In more recent studies surgeons have been more aggressive with the rehabilitation and allow partial weight bearing with some degree of motion early. There has been no evidence to support any post-operative regime; however given the similar failure rates, the senior author advocates early partial weight bearing and early passive range of motion exercises to aid ease of recovery. As previously discussed, meniscal healing can take 4–6 months, so patients should be advised to avoid impact loading, deep knee flexion or pivoting which may increase the risk of a re-tear.

### Augmentation

To further aid the healing of meniscal repairs, several studies have evaluated various techniques that act to augment the repair. Shelbourne and Heinrich [42] reviewed 332 patients who underwent ACL reconstructions with stable lateral meniscal tears which were either neglected or rasped/trephined and had a low re-operation rate (2.4%) and suggested that the increased blood flow aided healing of the meniscus. Supervised neglect of stable medial meniscal tears also led to them remaining asymptomatic if just trephined/rasped during concomitant ACL reconstruction. Assessment of the repaired menisci was not performed and was deemed satisfactory if functional outcomes, radiographs were normal and if further surgery was avoided.

There have since been further studies which aim to promote healing including applying exogenous fibrin clots, stem cells, rasping and trephination or performing an ACL reconstruction which increases blood flow [43]. Most studies are used as a means to document technique rather than objectively assessing outcomes.

### Summary of outcomes

Despite there being a recent increase in the number of published series evaluating meniscal repairs, there is no consensus on the ideal technique. Grant et al. [44] performed a systematic review of 19 studies, to compare the outcomes of all-inside and inside-out techniques in isolated medial meniscal tears in the presence of an intact ACL at a mean follow-up of 38.1 months. They found a non-significant difference in clinical failure rate of 17% for inside-out repairs compared with 19% for pooled all-inside techniques. Although there were similar functional scores, there was a significantly higher rate of neurological injury in the inside-out repairs. It should be noted that there was a higher complication rate in the older generation of all-inside repairs which utilized rigid devices compared with the newer suture-based techniques. Nepple et al. [4] noted the increase failure rate from early to long-term, and performed a systematic review on meniscal repair outcomes with a minimum follow-up of 5 years (mean 7.4 years). They found, in their review of 13 studies, that there was a pooled failure rate of 23.1% with no significant difference between the different techniques. There was a small non-significant difference in failure rates for medial and lateral meniscal tears (24.2% and 20.2%, respectively). This may be because the medial side is more tightly attached to the tibial plateau and has to transmit higher loads; however noted that the studies that found a difference were heterogenous in patient and tear characteristics. As they expected, they found 30% of the failures occurred after 2 years. Whilst the trend in increasing failure rates were seen in all techniques, conclusions cannot be firmly drawn on newer suture-based all-inside techniques as there have been no long-term studies published yet. Additionally, in all studies there was large heterogeneity including tear pattern, location, patient demographics, and the authors stated that non-significant differences may be due to underpowered studies.

### Trends and factors associated with repair

In 2013, Abrams et al. [3] published their review in the trends of knee arthroscopic procedures performed between 2005 and 2011 using a national database compiled from a collection of private insurance records. There were 387,833 meniscectomies and 23,640 meniscal repairs performed. Whilst there was no increase found in the number of meniscectomies performed, there was a doubling of meniscal repairs performed (*p *= 0.01) over that time, suggesting that it is only now that meniscal repairs are preferentially performed over meniscectomies. Wyatt et al. [45] reviewed a large cohort (*n *= 5712) of patients with meniscal tears undergoing ACL reconstruction, and found significant factors which determined if the meniscus was repaired. These included younger patient age, lower BMI, higher surgical volume, and if the surgeon was sports fellowship trained. Patients aged 14–17 had the highest chance of meniscal repair, and for every year increase, there was a 4% decreased chance of meniscal repair. In their series, they found that the surgeons who had undergone sports fellowships were 33.6% likely to repair a meniscus compared with those who had not gone on a fellowship (19.8%).

### Partial meniscectomy versus repair

Although in recent years there has been a trend towards meniscal preservation and repair, there is very limited data that directly compare the two procedures, and there are no randomized controlled trials comparing them. Current studies suggest that meniscectomy predisposes patients to early onset degenerative changes and also that patients with a meniscal repair have a higher functional outcomes. Evaluating long-term outcomes of meniscal repair [26, 39, 40], degenerative changes are seen ranging from 14 to 28% at a pooled follow-up of 12.5 years; however, it is important to note that patients with failed repairs were often excluded from further evaluation, and in those patients degenerative changes can be seen in as many as 56–57% of patients [39, 40]. Petty and Lubowitz [46] performed a systematic review of 5 studies and found a significantly higher rate of degenerative changes in patients undergoing partial meniscectomy (up to 53%) compared with the contralateral uninjured knee; however clinical symptoms of osteoarthritis were not observed at follow-up ranging from 8 to 16 years.

Stein et al. [47] evaluated long-term functional outcomes in athletes and found that although there was no difference at midterm (3.43 years) follow-up with regard to return to sports, patients undergoing partial meniscectomy had significantly worse outcomes at long-term follow-up. They found that only 50% in the partial meniscectomy group were able to return to sports compared with 96.15% in the repair group at 8.8-year follow-up. It is important to note that patients in the partial meniscectomy group were 3 years older (34.8 cf 31.5 years) and this may have partly accounted for this difference. Furthermore, despite these gains in functional outcomes, many studies exclude failed repairs from their evaluations. Paxton et al. [48] found in their systematic review of re-operation rates that patients undergoing meniscal repair were much more likely to have further procedures compared with patients undergoing meniscectomy at early and long-term follow-up (1.4% vs. 16.5% and 3.9% vs. 20.7%, respectively). Fortunately, it appears that the amount of meniscectomy is rarely increased when compared to the initial lesion after a failed repair [23].

Whilst there is undeniable evidence that the menisci aids in femoral contact area and load distribution the amount of required meniscal tissue is still debated. Although Lee et al. [49] found large decreases in contact area and increases in tibiofemoral stresses when the amount of meniscal resection increased from 50% to segmental/total, there were little changes when the amount of meniscal resection is small (20–30%) [50]. Furthermore, as meniscal repair tends to have a much longer rehabilitation period compared with meniscectomy, a failed repair would have cause more disturbance to a sporting season. Patients should therefore be counseled about the high re-operation rate.

## Conclusions

In summary, the number of meniscal repairs has been increasing in recent years due to the increasing awareness of the importance of meniscal preservation and associated early degenerative changes associated with meniscectomy. Accompanying this, the indications for meniscal repair have now expanded and include patients of any age provided they are active, and may be amenable even if the tear extends to the avascular zone. There is no difference in failure rates for any technique; however there are currently no long-term published studies on the newer suture-based all-inside devices. Patients should be counseled on the high rate of re-operation. There is very limited evidence that directly compares meniscectomy to meniscal repair, which may be due to the variability in tear characteristics, patient factors and rehabilitation regimes.
